# Addressing Opioid-Related Chemical Coping in Long-Term Opioid Therapy for Chronic Noncancer Pain: A Multicenter, Observational, Cross-Sectional Study

**DOI:** 10.3390/jcm7100354

**Published:** 2018-10-14

**Authors:** Anyela Marcela Castañeda, Chang-Soon Lee, Yong-Chul Kim, Dasom Lee, Jee Youn Moon

**Affiliations:** 1 Department of Anesthesiology and Pain Medicine, Seoul National University Hospital College of Medicine, 101Daehak-ro, Jongno-gu, Seoul 03080, Korea; anymarcey@hotmail.com (A.M.C.); iparid@gmail.com (C.-S.L.); pain@snu.ac.kr (Y.-C.K.); 2 Department of Psychiatry, Seoul National University Hospital College of Medicine, 101 Daehak-ro, Jongno-gu, Seoul 03080, Korea; ds.elena.lee@gmail.com; 3 Department of Integrated Cancer Care Center, Seoul National University Cancer Hospital, 101 Daehak-ro, Jongno-gu, Seoul 03080, Korea

**Keywords:** chronic noncancer pain, opioids, opioid use disorder, chemical coping, frequency, long-term opioids, risk factors

## Abstract

Opioid consumption has increased worldwide, which carries the risk of opioid use disorder (OUD). However, the literature on OUD and opioid-related chemical coping (OrCC) in chronic noncancer pain (CNCP) is heterogeneous, with most studies conducted in the United States. We performed a multicenter, observational, cross-sectional study to address OrCC in long-term opioid therapy (LtOT) for CNCP in South Korea. The objectives were to determine the frequency and predictors of OrCC. We included 258 patients. Among them, fifty-five (21%) patients showed OrCC. The sample had high pain catastrophizing (≥30 points; 66%), moderate-severe insomnia (≥15 points; 63%), low resilience (68 points), and high suicidal ideation (67%). OrCC patients had greater pain interference (85.18% vs. 58.28%, *p* = 0.017) and lower satisfaction with the LtOT (56.4% vs. 78.3%, *p* = 0.002). In multivariable analysis, alcohol abuse (OR = 6.84, *p* = 0.001), prescription drugs abuse (OR = 19.32, *p* = 0.016), functional pain (OR = 12.96, *p* < 0.001), head and neck pain (OR = 2.48, *p* = 0.039), MEDD (morphine equivalent daily dose) ≥ 200 mg/day (OR = 3.48, *p* = 0.006), and ongoing litigation (OR = 2.33, *p* = 0.047) were significant predictors of OrCC. In conclusion, the break-out of OrCC in CNCP in South Korea was comparable to those in countries with high opioid consumption, such as the United States, regardless of the country’s opioid consumption rate.

## 1. Introduction

Chronic pain is a devastating disease that is often treated inadequately [1]. Among a plethora of treatments, opioid agonists are one pharmacotherapy for moderate-severe pain. Although its consumption by country (mg/capita) has increased in the last two decades [2,3,4], it may remain under-requirements for managing moderate-severe pain in some regions, including Asian countries [1,4,5]. According to the 2015 opioid consumption data, the medical opioid consumption in the United States (U.S.) was 678 mg/capita while in South Korea (S. Korea), it was 55 mg/capita which was below average, ranking 43rd globally and 30th among thirty-five Organisation for Economic Cooperation and Development (OECD) countries (258 mg/capita average in OECD countries) [4]. However, it is remarkable that the opioid consumption in S. Korea has increased 5–6 times since 2005 (10 mg/capita), ranking 3rd among Asian countries preceded only by Vietnam (62 mg/capita) and Malaysia (60 mg/capita).

Many clinicians are reluctant to prescribe opioids due to the risk of opioid use disorder (OUD) secondary to the induced reward responses to the drug [6]. In chronic noncancer pain (CNCP), concerns regarding drug dependence from long-term opioid therapy (LtOT), increased all-cause mortality, and poor long-term treatment results in terms of pain relief and quality of life have made the use of opioids controversial [7,8,9,10]. The spectrum of OUD in CNCP is wide and varies from opioid abuse to addiction [11]. Between these extremes, opioid-related chemical coping (OrCC) is the use of opioids to cope with emotional distress characterized by inappropriate and/or excessive opioid use [12]. OrCC should be distinguished from addiction, a brain disease that involves neuroplasticity and substantial loss of self-control [13]. All addicts are chemical copers, but not all chemical copers are addicts [11]. Although OrCC was first defined in cancer patients [14], the correlation with OUD in CNCP patients is high [15]. Therefore, understanding this intermediate status may prompt the identification of risk factors for severe OUD and prevention of unnecessary opioid toxicity [16].

The literature on OUD and OrCC is heterogeneous, and an overwhelming majority of the studies took place in the U.S. [7,17,18,19], a country with high opioid consumption rates [5] and a current opioid epidemic. In the U.S., drug overdose deaths (the majority involving an opioid) have nearly quadrupled since 1999 [2], and growing evidence suggests no benefits of the LtOT over non-opioid therapy in CNCP patients [9]. However, despite the high consumption rates of opioids, other studies suggest that CNCP remains undertreated [1,20,21,22,23] and stringent regulations to prevent opioid abuse and addiction may result in inadequate pain control [24], especially in countries with low opioid consumption rates [4,5]. Moreover, it is uncertain if OUD is correlated with the country’s overall consumption rates of opioids (mg/capita). Consequently, it may be necessary to determine the frequency and characteristics of OUD in CNCP patients in countries with low-moderate opioid consumption rates.

Given the above, we performed a national, multicenter, observational study to address OrCC, the intermediate status of OUD, in LtOT for CNCP in S. Korea, a country with moderate opioid consumption rates [5]. The objectives of this study were to estimate the frequency of OrCC, to evaluate the patient’s functional and psychiatric characteristics, and to determine the risk factors associated with OrCC.

## 2. Materials and Methods

This national, observational, cross-sectional study was conducted in eight tertiary university-based hospitals in S. Korea between April 2017 and January 2018. The study was conducted in accordance with the Declaration of Helsinki, and the protocol was approved by the Institutional Review Boards (IRB) in each hospital. The protocol of the study was registered and openly shared in ClinicalTrials.gov (NCT03161795) to stress in the transparency of the research conduction. Eleven pain specialists, one neuropsychiatrist, and one oncologist took part in the study. Written informed consent was obtained from each participant prior enrollment. All methods and results have been reported according to the STROBE recommendations [25].

### 2.1. Study Participants

Inclusion criteria: (1) age ≥18 years; (2) diagnosis of chronic pain defined by the International Association for the Study of Pain (IASP) as persistent or recurrent pain lasting longer than 3 months or past the time of normal tissue healing [26]; (3) patients with LtOT defined as the current and regular use of one or more opioid prescriptions for ≥3 months; and (4) patients who completed the questionnaires administered in the study.

Exclusion criteria: (1) patients with a cancer diagnosis and/or receiving ongoing cancer treatment, palliative care, or end-of-life care; (2) patients who received opioid therapy for <3 months or intermittently; (3) patients with serious systemic diseases or acute psychiatric disorders that required inpatient management (schizophrenia, anxiety, depression, etc.), which compromised their safety or the completion of the study; or (4) patients with intellectual impairment and unable to answer the survey questions.

### 2.2. Evaluation of Opioid-Related Chemical Coping

The evaluation of OrCC was discussed by eleven anesthesiologists, one neuropsychiatrist, and one oncologist in the initial expert meeting. The presence of OrCC was determined through a questionnaire that contained seven behaviors related to OrCC. The questionnaire was based in a previous study of OrCC [12] and the DSM-5 diagnostic criteria of OUD [27] (Table 1). Two or more affirmative answers to the questionnaire were considered positive for OrCC. The questionnaire was reviewed through two additional educational meetings that were held prior to the patient’s enrollment to reduce bias between physicians. A pain specialist at each participating hospital evaluated the presence of OrCC, thus the self-administered chemical coping inventory (CCI) was not considered for this study [11].

### 2.3. Outcome Measurements

Patients’ sociodemographic data were obtained from the electronic medical record (EMR) including educational level (<high school or ≥high school) and religion (yes = Christianity, Islam, Buddhism, Hinduism, Taoism, etc.; no = Atheism), pain characteristics (including pain intensity using an 11-point numerical rating scale (NRS)) [28], co-morbid psychopathologies, substance abuse history within 1 year, and secondary morbid gain (if the patient’s pain allows him/her to miss work, avoid military duty, obtain financial compensation, obtain drugs, etc.). We also collected opioid information, which included the duration of administration, opioid name and type, administration route, morphine equivalent daily dose (MEDD, mg/day) [29], initial prescribers, number of opioid-seeking medical/emergency room visits per year, and co-prescription of benzodiazepines or other medication. The information that was unavailable in the EMR was asked directly to the patient when appropriate. The tools and questionnaires administered in this study were divided into patient’s and physician’s booklets. The physician’s booklet included a questionnaire to assess the patient’s OrCC (Table 1) and the patient’s booklet contained predictive tools for OUD and questionnaires to address functionality.

The risks of LtOT were assessed through a survey in the outpatient setting of each pain clinic. After obtaining written, informed consent, the patients received a patient’s booklet and responded to the following questionnaires and forms: (1) Cut down, Annoyed, Guilty, Eye-opener Adapted to Include Drugs (CAGE-AID) [30]; (2) Brief Pain Inventory-Short Form (BPI-SF) [31]; (3) Pain Catastrophizing Scale (PCS) [32]; (4) Hospital Anxiety and Depression Scale (HADS) [33]; (5) Insomnia Severity Index (ISI) [34]; (6) Korean Instrumental Activities of Daily Living Scale (K-IADL) [35]; (7) Korean Connor-Davidson Resilience Scale (K-CD-RISC) [36]; and (8) Patient Global Impression of Change Scale (PGIC) [37]. Among the four questions in the CAGE-AID, one or more affirmative answers was considered “positive” for OUD [38]. BPI-SF measured pain intensity (Items 3–6) and pain interference (Item 9) [39], which had seven components scored from 0 (no interference) to 10 (interferes completely). PCS had 13 items rated from 0 (not at all) to 4 (all the time); a total score ≥30 was considered “catastrophizing” [32]. HADS scores for anxiety and depression ranged from 0 to 21, with ≥11 points considered “abnormal” [40]. The ISI total score ranged 0–28; scores ranging from 15–21 and 22–28 indicated moderate and severe insomnia, respectively [41]. K-IADL evaluated daily activities with 11 questions rated from 0 (independently performed/normal) to 3 (impossible to perform) [42]. K-CD-RISC had 25 items, rated from 0–4, with higher scores reflecting greater resilience [43]. PGIC was rated from 1 (very much improved) to 7 (very much worse) [44]. Patients’ overall satisfaction with their LtOT ranged from 1 (extremely satisfied) to 5 (extremely unsatisfied). A question to evaluate the presence of suicidal ideation in CNCP was also included (yes = previous suicidal attempts, thoughts of ending one’s life, planned to commit suicide, wish to be dead; no = never attempted or thought about committing suicide). Additionally, adverse and undesirable effects of opioids were collected.

On the survey day, after answering the patient’s booklet, each patient attended a routine visit with a pain specialist. Once the patient exited the room, the specialist answered the questionnaire to assess the patient’s OrCC (Table 1) included in the physician’s booklet.

### 2.4. Sample Size Calculation and Statistical Analysis

The precision/absolute error and the significance level were set at 5% and 95%, respectively (Type 1 error of 5%, α = 0.05). According to a published study by Kwon et al. [17], the prevalence of chemical coping was approximately 18%; therefore, the sample size was calculated to be 235 participants. Considering a 10% dropout rate, a group of 258 participants was planned for recruitment.

Depending on the data distribution, independent *t*-tests or Wilcoxon rank sum tests were performed to compare two independent groups. A paired *t*-test was used to compare two means from the same group. Categorical data were analyzed using Pearson’s χ^2^ test, Fischer’s exact test or Chi-square test. The normality distribution for continuous variables was assessed with the Kolmogorov–Smirnov test. The independent *t*-test was used to compare normal distribution and the Mann–Whitney U test was used for non-normal distribution.

Univariable analysis was performed to explore variables associated with OrCC, using the presence of OrCC as a dependent variable and clinical variables that included sociodemographic data, pain characteristics, opioid information, scores of CAGE-AID, K-IADL, PCS, and BPI-SF, as independent variables. Clinical variables with a *p*-value < 0.1 in univariable analysis were considered for multivariable analysis. The multivariable regression analysis was conducted by manual forward stepwise selection, and variables with a *p*-value < 0.05 were retained.

All parametric data were presented as mean ± standard deviation (SD) and nonparametric data as percentage (%) or odds ratio (OR) with a 95% confidence interval (95% CI). All *p*-values are two-tailed, and *p*-values < 0.05 were considered statistically significant. Statistical analyses were performed using SPSS version 22.0. (IBM Corp., Armonk, NY, USA).

## 3. Results

**Figure 1:**
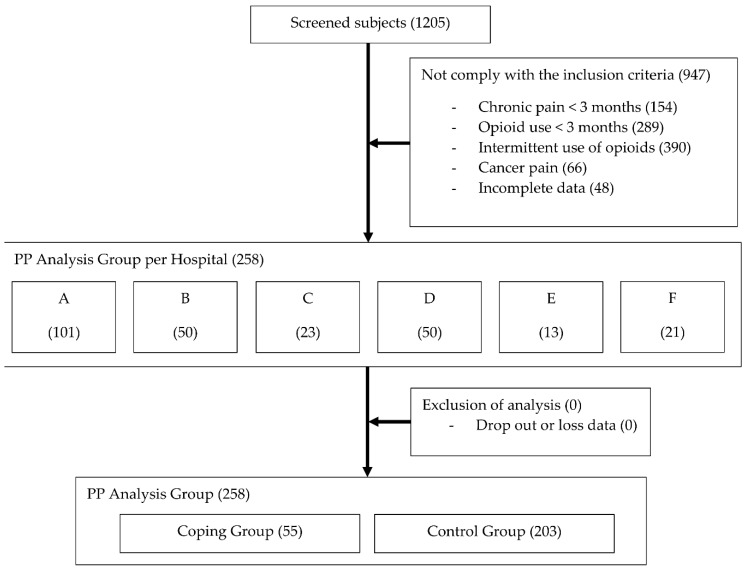
Flow diagram of participants. A, B, C, etc., indicate the hospitals that participated in the study. PP, per-protocol.

A total of 258 CNCP patients receiving LtOT, in six of eight hospitals, were included in the study (Figure 1). Patients from two hospitals were excluded due to delayed IRB approval. Based on the pre-defined consensus, 55 patients (21%) were classified as OrCC.

The patients were divided into two groups according to a positive OrCC (the coping group (*n* = 55) and control group (*n* = 203)). Table 2 demonstrates the patients’ sociodemographic data and clinical characteristics. The sample was homogenous in terms of ethnicity, sex, BMI, marital status, employment, and religion. The average pain duration was 74.55 months (95% CI: 66.68–82.43). When compared to the control group, patients in the coping group were younger (48.58 ± 12.25 vs. 53.79 ± 13.54; *p* = 0.038) and with an education level greater or equal to high school level (90.9% vs. 73.9%; *p* = 0.007). Although the reduction of NRS pain score from the initial to final visit was significant within each group (*p* < 0.001 in controls and *p* = 0.048 in copers), it was less than 1 point in both groups. Pain in the head and neck, functional pain syndrome, and mixed pain were more common in copers (27.3% vs. 13.3%, *p* = 0.013; 18.2% vs. 2.5%, *p* < 0.001; and 18.2% vs. 8.4%, *p* = 0.035, respectively). Alcohol and/or medication abuse, and prescription drug use with alcohol within one year, were remarkably frequent in copers when compared to non-copers (20.0% vs. 3.9%, *p* < 0.001; 9.1% vs. 0.5%, *p* < 0.001; and 22.6% vs. 8.5%, *p* = 0.02, respectively). More copers had co-morbid depression (50.9% vs. 27.6%, *p* = 0.001) and reported ongoing litigation (27.8% vs. 13.9%, *p* = 0.010). Additionally, an overwhelming 66.7% of the sample (*n* = 172) had suicidal ideation related to their chronic pain.

The opioid information is shown in Table 3. The duration of opioid administration and number of patients with co-prescription (including benzodiazepines) was not significantly different between groups. Although the opioid types (long-acting vs. short-acting) were similar in both groups, rapid-onset fentanyl and intravenous injections were more frequent in the coping group (14.5% vs. 3.4%, *p* = 0.005 and 10.9% vs. 3.0%, *p* = 0.023, respectively). The average MEDD (mg/day) was significantly higher in the copers than the non-copers (169 ± 186 vs. 119 ± 227, *p* = 0.006). Patients with MEDD ≥100 and ≥200 mg/day were more frequent in the coping group (32.7% vs. 21.2%, *p* = 0.033 and 25.5% vs. 9.9%, *p* = 0.002, respectively). The number of annual visits to an opioid prescriber and the number of patients who visited the ER seeking for opioids was significantly higher in the copers than non-copers (36.35 ± 53.93 vs. 19.07 ± 18.86 visits, *p* = 0.023 and 27.3% vs. 4.4%, *p* < 0.001, respectively). The first opioid prescriber was not significantly different between groups; and in 81% of the sample, the first opioid prescriber was a pain specialist.

Table 4 shows the questionnaires and predictive tools used in the study. Although the proportion of patients with a positive CAGE-AID was higher in the copers (80.0% vs. 66.5%), it did not reach statistical significance (*p* = 0.054). The PCS was over 30 in both groups, indicating a “catastrophic” appraisal of pain. The “worst” NRS item of the BPI-SF was higher, and the general activity, mood, and sleep interference were worse in the copers than the non-copers (*p* = 0.001, *p* = 0.043, *p* = 0.013, and *p* = 0.021, respectively). The K-IADL score and percentages were higher in the coping group (*p* = 0.031 and *p* = 0.017, respectively). Both groups reported high anxiety and depression in HADS, moderate clinical insomnia in the ISI, and low resilience in the K-CD-RISC.

About 74% of the subjects were extremely or somewhat satisfied with their LtOT, and the percent of patients unsatisfied was significantly more prevalent among copers vs. non-copers (*n* = 24, 44% vs. *n* = 44, 22%; *p* = 0.002). The PGIC was similar in both groups. There were no differences in the adverse or undesirable effects between groups, and 62% of the patients reported at least one event. The most frequent was constipation (*n* = 105, 40.7%) followed by somnolence (*n* = 62, 24.0%) and nausea (*n* = 50, 19.4%).

**Figure 2:**
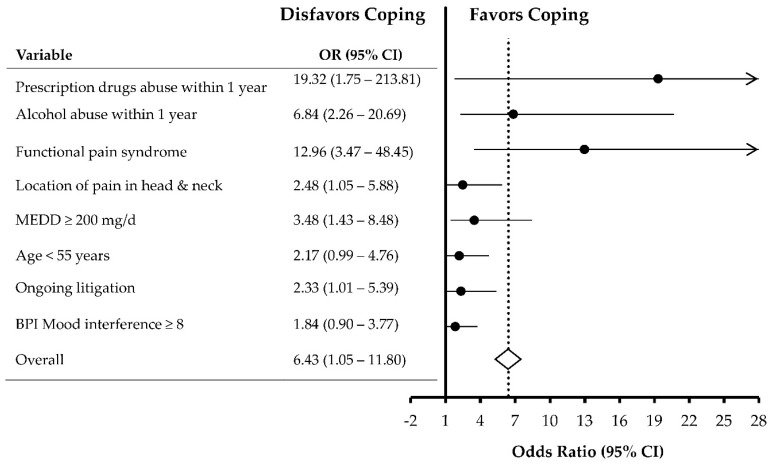
Forest plot of multivariable analysis showing the factors independently associated with opioid-related chemical coping. OR, odds ratio; CI, confidence interval; BPI, brief pain inventory; MEDD, morphine equivalent daily dose.

Figure 2 shows the independent predictors of OrCC identified in multivariable analysis. The risk of OrCC increased in patients with: (1) prescription drugs abuse (Odds ratio (OR) = 19.32 (95% confidence interval (CI) = 1.75–213.81), *p* = 0.016); (2) alcohol abuse (OR = 6.84 (95% CI = 2.26–20.69), *p* = 0.001); (3) functional pain syndrome (OR = 12.96 (3.47–48.45), *p* < 0.001); (4) head and neck pain (OR = 2.48 (95% CI = 1.05–5.88), *p* = 0.039); (5) MEDD ≥ 200 mg/day (OR = 3.48 (95% CI = 1.43–8.48), *p* = 0.006); and (6) ongoing litigation (OR = 2.33 (95% CI = 1.01–5.39) *p* = 0.047). Additionally, age < 55 years (OR = 2.17 (95% CI = 0.99–4.76), *p* = 0.052) and BPI-SF mood interference ≥ 8 (OR = 1.84 (95% CI = 0.90–3.77), *p* = 0.096) remained in the multivariable model.

## 4. Discussion

This study evaluated the rate of OrCC and patient characteristics in a group of CNCP patients receiving LtOT. The frequency of OrCC was 21%, which indicates that about one out of every five CNCP patients used opioids to cope with emotional distress. There is a scarcity of research regarding the frequency of OrCC, with the except of one study [17] which reported a rate of 18% in palliative care patients in the U.S. Therefore, to the best of our knowledge, this is the first study to evaluate the rate of OrCC in CNCP. Our results demonstrate that the frequency of CNCP patients coping chemically with opioids is as high as that found in cancer patients [17]. Furthermore, it is comparable to the rate of misuse (21–29%) determined in a recent systematic review that included 35 studies from the U.S. and three studies from the European Union (EU) [19]. Our results show that OrCC in CNCP is comparably high to OUD rates, even in countries with low-moderate opioid consumption.

Regarding patients’ demographics, previous studies reported that young age and male sex are common risk factors for OUD and dependency [45,46]. In this study, although the copers were younger, patient sex was not statistically significant, which correlates with another OrCC study [17]. OrCC patients had high level of education compared to the non-copers, which contradicts previous studies in substance abuse and dependence [47,48]. The discrepancy in our finding may be explained as an interaction effect between age and level of education (correlation coefficient = −0.178, *p* = 0.005). In our study, younger patients had higher level of education and conversely older patients had lower than high school education level. A recent report found that 66% of Koreans, between the age of 25 and 34 years, attained tertiary education, while only 8% of Korean women aged 55–64 years did it [49]. Therefore, younger patients with an increased liability to OrCC had higher education levels, which may explain our results.

In terms of the pain characteristics, the overall patients in this study complained of moderate to severe pain with the NRS pain score of >7 at their initial visits. Despite LtOT, however, their pain improvement on the last measurement was trivial with only 8.6% reduction in the pain severity. In addition, the prevalence of posttraumatic stress disorder in the study’s sample was relatively high (*n* = 52, 20.2%) without significant differences between the groups. Those patients have more risk factors for pain, including higher rates of psychiatric and substance use disorders [50], which may explain the high frequency of the disorder found in this study. Another interesting result in this study was that head and neck pain increased 2.5 times (*p* = 0.039), and functional pain disorders increased 13 times the risk of OrCC (*p* < 0.001) in our multivariable analysis. Functional pain syndromes typically concur with anxiety, depression, and chronic fatigue syndrome [51], conditions for which opioids are usually ineffective [52]. Therefore, the treatment of chronic functional pain should be centered in non-opioid pharmacotherapy with active use of physiotherapeutic and psychological methods to improve coping with pain [53]. Moreover, patients receiving LtOT without improvement in the pain control should be evaluated to assess the real contribution of opioids and to reduce drug toxicity.

Major depression and alcohol or drug abuse are known risk factors for OUD and OrCC [16,54,55] which is concordant with our result. Markou et al. [56] asserted that depression has neurobiological effects similar to those in alcohol or opiate withdrawal syndromes. Hence, patients with underlying depression may self-medicate with opioids to correct their dysfunctional systems. Our sample had high HADS scores without statistically significant differences between groups, which may be explained by the scale’s low specificity (~50%) and sex/age-related biases [57], and due to the high prevalence of anxiety and depression in chronic pain patients with LtOT. In addition, alcohol and prescription drug abuse also increased 7 (*p* = 0.001) and 19 times (*p* = 0.016) the risk of OrCC in our results. The concomitant use of alcohol and opioids is associated with OUD, OrCC and worse outcomes [58], which is consistent with our result (*p* = 0.002). Therefore, CNCP patients with alcohol and/or prescription drug abuse history require special attention due to an increased risk of OrCC, opioid toxicity, and poor outcomes.

Similar to previous studies in OUD [59,60], patients with OrCC received significantly higher dosages of opioids (*p* = 0.006) in this study. Interestingly, doses of 100–200 mg/day were not different among the groups (*p* = 0.878). However, dosages ≥200 mg/day almost quadrupled the risk of OrCC (*p* = 0.002). Another study from the U.S. also found increased OUD rates with dosages ≥200 mg/day, without differences at 100 or 120 mg/day [61]. Therefore, dosages ≥200 mg/day should be concerning in CNCP due to a high correlation with OrCC and OUD. In terms of opioid types, rapid-onset opioids (ROOs) were prescribed more frequently in the coping group. ROOs are used for the management of breakthrough pain (BTP) in opioid-tolerant patients with cancer or noncancer pain [62,63,64]. Although the evidence linking ROOs to OUD is limited [65,66], our results support that ROOs may potentiate OUD. A cautious use of ROOs in CNCP patients is recommended and further studies that evaluate its association with OUD are needed. Additionally, frequent visits to the provider and/or the ER seeking for opioids was correlated with OrCC (*p* = 0.001 and *p* < 0.001 respectively). Therefore, although pseudo-addiction should be initially discarded as a cause of opioid seeking [67], frequent hospital and ER visitors must be evaluated for OUD.

The BPI-SF showed increased pain interference, and the K-IADL indicated an increased compromise of daily activities in the copers. Our results suggest that decreased functionality and high pain interference constitute risk factors of OrCC [68,69]. Ongoing litigation doubled the risk of OrCC. Although previous studies have not linked litigation with OUD, this process causes negative emotions that accentuate the underlying pain with anger, frustration, and helplessness [70], which may induce OrCC. Furthermore, two-thirds of the sample had catastrophic thinking and moderate-severe insomnia. Pain catastrophizing is associated with pain severity, altered CNS pain processing, and exaggerated pain-related interference [71]. Our sample had low resilience (68/100 points), compared to the U.S. general population average (80/100 points) [35]. These findings highlight the role of psychological therapy in improving pain-coping skills and functionality in CNCP patients [72,73].

Contrary to previous studies on chemical coping [17,67], in this study, the CAGE-AID questionnaire was not significantly positive in the OrCC group when compared to the controls (*p* = 0.054). Interestingly, CAGE-AID positives were found in 66.5% of the controls, whereas 20% negatives were copers. This result infers that CAGE-AID is a predictive, but not a diagnostic tool for OUD with a low specificity [17,74]. Moreover, the questionnaire focuses on addiction and may not detect risky use in non-dependent individuals [30], as in our study population. Another distinctive result is that pain specialists were the predominant opioid prescribers. S. Korea’s strict regulations on opioids and the difficulties of storage and administration limit their use by primary specialists [75]. Conversely, in the U.S., the primary care specialty groups accounted for nearly half (44.5%) of all dispensed opioid prescriptions during 2007–2012 [76]. Additionally, insufficient training in the management of CNCP and excessive focus on the treatment with opioids may lead to its over-prescription and the under-detection of OUD [77,78]. Accordingly, the mean amount of opioids prescribed per person in 2015 in the U.S. was 640 mg/day (0.1–5543 mg/day) [2], almost five times the mean in our study 129 mg/day (4.5–2700 mg/day).

Another remarkable finding in this study is the absence of illicit drug abuse reports. This result may be secondary to deep-rooted cultural and social stigmatization of illicit drugs in Asia [79]. Historically, S. Korea has been viewed as a drug-free country when compared to the U.S., Japan, and other countries [80]. Traditional drugs, including heroin and cocaine, are not commonly used in S. Korea, as reflected by drug seizure and arrest data [81]. Consequently, the laws that control illicit drug-use may influence the rates of overdose deaths in S. Korea.

There are several limitations to be addressed. First, this study took place only in tertiary hospitals. This may be associated with biases for generalization since the patients in this study may have more challenging pain syndromes than those in primary institutions. Second, the questionnaire used to evaluate OrCC was a result of an expert meeting, however, it is not a validated tool. In addition, although there were three consensus and educational meetings prior to patient enrollment, there might be detection biases between pain specialists. Nonetheless, OrCC is a clinical phenomenon accurately assessed by experienced providers [14], thus, a high predictability of true positives may be expected. Moreover, in this study, OrCC was evaluated immediately after each visit to avoid inappropriate scoring or recall biases. Third, our sample size was relatively large (*n* = 258); however, a broad CI of some OrCC risk factors in our multivariable analysis, such as prescription drug abuse (OR = 19.32 (95% CI = 1.75–213.81)) or functional pain syndrome (OR = 12.96 (95% CI = 3.47–48.45)), would be a limitation. Another drawback is that the study’s data depended on statements from patients. Although there was assurance of confidentiality, patients’ responses may not always be reliable. Finally, urine drug test (UDT) and opiate immunoassay, which are considered “gold standards” to assess OUD [7], were not conducted. Barriers to cost-effectiveness and accessibility restricted their use in this study.

## 5. Conclusions

Approximately 21% of the CNCP patients receiving LtOT are chemically coping with opioids, carrying high intensity of pain, and experiencing severe interference in daily activities. The high rates of OrCC found in this study suggest that the break-out of OUD in CNCP of S. Korea is comparable to those in countries with high opioid consumption, such as the U.S., regardless of the country’s opioid consumption rates. Therefore, we should be vigilant about OUD in CNCP patients with LtOT. The independent risk factors of OrCC are prescription drugs and alcohol abuse, functional pain syndrome, pain in the head and neck, MEDD ≥ 200 mg/day, and ongoing litigation. Although further validation studies are warranted, the assessment of OrCC may prompt the identification of patients at high risk for severe OUD. Finally, although our result has suggested that there is no benefit of LtOT in CNCP, more research is needed to establish the rationale of evidence-based opioid prescription that should be limited to short-term use as much as possible.

## Figures and Tables

**Table 1:** Opioid-related chemical coping definition and questionnaire for physicians.

1. Please read carefully the definition of opioid-related chemical coping: “Opioid-related chemical coping is the use of opioids to cope with emotional distress characterized by inappropriate and/or excessive opioid use” [12].	
2. The following are aberrant behaviors related to chemical coping with opioids. Please mark all the behaviors which you believe the patient presents:	
**Behavior**	**Check**
Use of opioids other than for the prescribed purpose to treat non-nociceptive symptoms (cope with emotional or spiritual distress, anxiety, depression, insomnia, fatigue, anger, etc.). Excessive use (more than prescribed according to appropriate titration) of PRN (pro re nata) doses despite no benefits being added to pain relief or quality of life. The patient has obtained or stole prescription opioids from another person (family member, friend, etc.). The patient asks the physician to prescribe a specific opioid or certain amount of the opioid. Impulsive or excessive use of the prescribed opioids despite several and persistent secondary effects (drowsiness, nausea, vomiting, constipation, etc.). The patient has insisted aggressively to receive higher doses of an opioid for storage purposes, prevention, fear, etc.. The patient keeps losing the prescription of opioids and often seeks to visit the opioid provider to get new prescriptions and feel reassured.	







**Table 2:** Demographic variables and clinical characteristics.

Variable	Overall (*n* = 258)	Control (*n* = 203)	Coping * (*n* = 55)	*p*-Value
Gender, *n* (%) Male Female	153 (59.3) 105 (40.7)	120 (59.1) 83 (40.9)	33 (60.0) 22 (40.0)	0.905
Age, mean ± SD, years	52.89 ± 3.36	53.79 ± 13.54	48.58 ± 12.25	0.038
Ethnicity, *n* (%), Asian	258 (100)	203 (78.7)	55 (21.3)	-
BMI, mean ± SD, kg/m^2^	24.81 ± 4.03	24.89 ± 3.87	24.51 ± 4.58	0.544
Marital status, *n* (%) Married Single Divorced/Widowed	64 (24.9) 185 (72.0) 8 (3.1)	50 (24.8) 146 (72.3) 6 (3.0)	14 (25.5) 39 (70.9) 2 (3.6)	0.960
Education level, *n* (%) <high school ≥high school	58 (22.6) 200 (77.5)	53 (26.1) 150 (73.9)	5 (9.1) 50 (90.9)	0.007
Employment status, *n* (%) Unemployed (students and housewives included) Employed	192 (75.6) 62 (24.4)	152 (75.6) 49 (24.4)	40 (75.5) 13 (24.5)	0.982
Religion, *n* (%) No Yes	130 (50.6) 127 (49.4)	106 (52.5) 96 (47.5)	24 (43.6) 31 (56.4)	0.245
Chronicity of pain, mean ± SD, months	74.55 ± 64.25	73.23 ± 66.09	79.44 ± 57.23	0.526
NRS, mean ± SD, points Initial Current Absolute change *p*-value of absolute change Percent change	7.38 ± 1.61 6.55 ± 2.09 −0.83 ± 2.29 <0.001 −8.6 ± 32.3	7.37 ± 1.54 6.51 ± 2.10 −0.89 ± 2.41 <0.001 −8.9 ± 22.1	7.43 ± 1.89 6.86 ± 2.07 −0.58 ± 1.74 0.048 −8.5 ± 34.4	0.730 0.309 0.364 0.936 ^†^
Etiology of pain, *n* (%) Trauma Surgery Degenerative Disease Combined Idiopathic	128 (49.6) 51 (19.8) 15 (5.8) 73 (26.7) 1 (0.4) 9 (3.5)	96 (47.3) 43 (21.2) 10 (4.9) 56 (28.1) 1 (0.5) 6 (3.0)	32 (58.2) 8 (14.5) 5 (9.1) 17 (32.7) 0 (0.0) 3 (5.5)	0.152 0.273 0.325 ^‡^ 0.236 1 0.407 ^‡^
Location of pain, *n* (%) Head and Neck Chest or Abdomen Back Extremities Others^§^ or unknown	42 (16.3) 35 (13.6) 102 (39.5) 197 (76.4) 20 (7.8)	27 (13.3) 24 (11.8) 77 (37.9) 155 (76.4) 15 (7.4)	15 (27.3) 11 (20.0) 25 (45.5) 42 (76.4) 5 (9.1)	0.013 0.116 0.311 0.999 0.776
Type of pain, *n* (%) Nociceptive Neuropathic Functional Mixed	36 (14.0) 197 (76.4) 15 (5.8) 27 (10.5)	29 (14.3) 160 (78.8) 5 (2.5) 17 (8.4)	7 (12.7) 37 (67.3) 10 (18.2) 10 (18.2)	0.767 0.074 <0.001 ^‡^ 0.035
Substance abuse history within 1 year, *n* (%) Yes Tobacco Alcohol Medication Illicit drugs Multiple	79 (30.6) 62 (24.0) 19 (7.4) 6 (2.3) 0 (0.0) 1 (0.4)	52 (25.6) 46 (22.7) 8 (3.9) 1 (0.5) 0 (0.0) 1 (0.5)	27 (49.1) 16 (29.1) 11 (20.0) 5 (9.1) 0 (0.0) 0 (0.0)	0.001 0.322 <0.001 <0.001 - 1.00 ^‡^
Taken prescription drugs with alcohol within 1 year, *n* (%)	29 (11.2)	17 (8.5)	12 (22.6)	0.002
Concurrent psychopathology, *n* (%) Yes Depression Anxiety PTSD Bipolar disorder Others	120 (46.5) 84 (32.6) 25 (9.7) 52 (20.2) 6 (2.3) 22 (8.5)	86 (42.4) 56 (27.6) 19 (9.4) 37 (18.2) 4 (2.0) 18 (8.9)	34 (61.8) 28 (50.9) 6 (10.9) 15 (27.3) 2 (3.6) 4 (7.3)	0.008 0.001 0.73 0.138 0.611 ^‡^ 1.00 ^‡^
Secondary morbid gain, *n* (%) Miss work or studies Avoid military duty Ongoing litigation	42 (53.1) 43 (16.7)	30 (83.3) 46 (22.8) 28 (13.9)	12 (92.3) 7 (13.0) 15 (27.8)	0.658 0.157 0.010
Suicidal ideation, *n* (%)	172 (66.7)	132 (65.3)	40 (75.5)	0.161

* The presence of OrCC was evaluated by a physician, using a questionnaire that contained seven behaviors related to OrCC. Two or more affirmative answers to the questionnaire were considered positive for OrCC. ^†^ Values from Mann–Whitney U test. ^‡^ Values from Fisher’s exact test. ^§^ Whole body or genitalia. BMI, body mass index; NRS, numerical rating scale; PTSD, post-traumatic stress disorder; SD, standard deviation.

**Table 3:** Opioid-related information.

Variable	Overall (*n* = 258)	Control (*n* = 203)	Coping * (*n* = 55)	*p*-Value
Duration of opioids, mean ± SD, months >12 months, *n* (%)	16.34 ± 31.08 65 (25.2)	15.90 ± 28.76 51 (25.1)	17.85 ± 38.25 14 (25.5)	0.722 0.747
Opioid types, *n* (%) Long-acting Oral long-acting Transdermal patch Short-acting Oral short-acting Rapid onset fentanyl Intravenous	231 (89.5) 213 (82.6) 85 (32.9) 145 (56.2) 141 (54.7) 15 (5.8) 12 (4.7)	184 (90.6) 170 (83.7) 63 (31.0) 109 (53.7) 107 (52.7) 7 (3.4) 6 (3.0)	47 (85.5) 43 (78.2) 22 (40.0) 36 (65.5) 34 (61.8) 8 (14.5) 6 (10.9)	0.265 0.335 0.210 0.119 0.229 0.005 0.023
MEDD, mean ± SD, mg/day ≥100 mg/day, *n* (%) ≥200 mg/day, *n* (%)	129 ± 220 95 (36.8) 34 (13.2)	119 ± 227 68 (33.5) 20 (9.9)	169 ± 186 27 (49.1) 14 (25.5)	0.006 ^†^ 0.033 0.002
Number of visits per year to the opioid provider, mean ± SD	22.77 ± 30.71	19.07 ± 18.86	36.35 ± 53.93	0.023
ER visits seeking opioids, *n* (%)	24 (9.3)	9 (4.4)	15 (27.3)	<0.001
First opioid provider, *n* (%) Family doctor General physician Surgeon ER physician Pain physician Others ^‡^ Unknown	2 (0.8) 6 (2.3) 20 (7.8) 2 (0.8) 209 (81.0) 18 (7.0) 1 (0.4)	1 (0.5) 5 (2.5) 13 (6.4) 2 (1.0) 166 (81.8) 15 (7.4) 1 (0.5)	1 (1.8) 1 (1.8) 7 (12.7) 0 (0.0) 43 (78.2) 3 (5.5) 0 (0.0)	0.702 0.609 0.778 0.120 0.460 0.547 0.617 0.602
Benzodiazepines, *n* (%)	120 (46.5)	95 (46.8)	25 (45.5)	0.859
Non-opioid medications, *n* (%) Antidepressants Anticonvulsants Topical agents	134 (51.9) 182 (70.5) 33 (12.8)	107(55.4) 149 (77.2) 24 (12.4)	27 (51.9) 33 (64.7) 9 (17.6)	0.651 0.068 0.333
Physical therapy, *n* (%)	32 (12.4)	28 (3.9)	4 (7.4)	0.203

* The presence of OrCC was evaluated by a physician, using a questionnaire that contained seven behaviors related to OrCC. Two or more affirmative answers to the questionnaire were considered positive for OrCC. ^†^ Values from Mann–Whitney U test. ^‡^ Gynecology, internal medicine, neurology, neuropsychiatry, orthopedics, otorhinolaryngology. ER, emergency room; MEDD, morphine equivalent daily dose; SD, standard deviation.

**Table 4:** Questionnaires and predictive tools.

Variable	Overall (*n* = 258)	Control (*n* = 203)	Coping * (*n* = 55)	*p*-Value
CAGE-AID, *n* (%) Negative Positive (≥1 positive)	79 (30.6) 179 (69.4)	68 (33.5) 135 (66.5)	11 (20.0) 44 (80.0)	0.106 0.054
PCS, mean ± SD, points ≥30 points, *n* (%)	34.22 ± 12.27 170 (65.9)	34.14 ± 12.33 134 (66.0)	34.51 ± 12.18 36 (65.5)	0.843 0.939
BPI-SF, mean ± SD, points Worst NRS NRS on average NRS right now Pain relief (%) Pain interference General activity Mood Walking ability Normal work Relations with other people Sleep Enjoyment of life	8.12 ± 1.97 6.63 ± 2.05 6.37 ± 2.36 48.44 ± 23.47 6.47 ± 2.48 6.59 ± 2.53 5.85 ± 3.14 6.38 ± 2.75 6.04 ± 3.26 6.29 ± 3.09 6.78 ± 3.00	7.95 ± 2.06 6.53 ± 2.05 6.29 ± 2.30 49.79 ± 22.39 6.31 ± 2.53 6.39 ± 2.56 5.77 ± 3.14 6.22 ± 2.79 5.88 ± 3.28 6.06 ± 3.12 6.66 ± 3.03	8.75 ± 1.42 6.98 ± 2.04 6.67 ± 2.58 43.45 ± 26.75 7.07 ± 2.20 7.35 ± 2.27 6.15 ± 3.15 6.96 ± 2.55 6.62 ± 3.15 7.15 ± 2.85 7.22 ± 2.85	0.001 0.152 0.288 0.112 0.043 0.013 0.437 0.076 0.137 0.021 0.221
K-IADL, mean ± SD, points Percentage	7.46 ± 7.18 64.01 ± 74.55	6.96 ± 6.90 58.28 ± 73.74	9.31 ± 7.90 85.18 ± 74.35	0.031 0.017
PGIC, *n* (%), better	108 (41.9)	89 (43.8)	19 (34.5)	0.215
Satisfaction scale, ^†^ Satisfied, *n* (%) Unsatisfied, *n* (%)	190 (73.6) 68 (26.4)	159 (78.3) 44 (21.7)	31 (56.4) 24 (43.6)	0.002
HADS Anxiety, mean ± SD, points ≥11 (abnormal), *n* (%) Depression, mean ± SD, points ≥11 (abnormal), *n* (%)	10.88 ± 4.99 125 (48.4) 11.76 ± 4.71 160 (62.0)	10.72 ± 4.80 96 (47.3) 11.74 ± 4.35 127 (62.6)	11.45 ± 5.66 29 (52.7) 11.80 ± 5.91 33 (60.0)	0.381 0.474 0.938 0.728
ISI, mean ± SD, points ≥15 (moderate-severe), *n* (%) ≥22 (severe), *n* (%)	16.83 ± 7.63 162 (62.8) 88 (34.1)	16.61 ± 7.62 124 (61.1) 66 (32.5)	17.62 ± 7.66 38 (69.1) 22 (40.0)	0.386 0.276 0.299
K-CD-RISC, mean ± SD, points	67.95 ± 22.06	68.77 ± 22.24	64.91 ± 21.30	0.250

* The presence of OrCC was evaluated by a physician, using a questionnaire that contained seven behaviors related to OrCC. Two or more affirmative answers to the questionnaire were considered positive for OrCC. ^†^ Satisfied = extremely satisfied and somewhat satisfied, unsatisfied = somewhat unsatisfied and extremely unsatisfied; BPI-SF, brief pain inventory-short form; CAGE-AID, cut down, annoyed, guilty, eye-opener—adapted to include drugs; HADS, hospital anxiety and depression scale; ISI, insomnia severity index; K-IADL, Korean-instrumental activities of daily living; K-CD-RISC, Korean-Connor-Davidson resilience scale; PCS, pain catastrophizing scale; PGIC, patient global impression of change; SD, standard deviation.
